# Erratum: Anwendungsbegleitende Erfolgsmessung (AbEM) bei Digitalen Gesundheitsanwendungen: Optimierung aus Sicht der Versorgungsforschung für eine zielgerichtete und versorgungsrelevante Erfolgsmessung

**DOI:** 10.1055/a-2963-7026

**Published:** 2026-10-13

**Authors:** Ursula Kramer, Theresa Sophie Busse, Nikolas Dietzel, Ria Heinrich, Pascal Kemmerer, Anna Niemeyer, Jutta G. Richter, Susanne Singer, Michael Zeiler, Madlen Scheibe

**Affiliations:** 1 GesundheitskommunikationSanaworkFreiburg im BreisgauBaden-WürttembergGermany; 2 Department für Humanmedizin, Juniorprofessur für Digital Health 235785 Universität Witten/Herdecke Fakultät für GesundheitWitten/HerdeckeGermany; 3 Interdisziplinäres Zentrum für Health Technology Assessment (HTA) und Public Health (IZPH)Friedrich-Alexander-Universität Erlangen-Nürnberg (FAU)ErlangenGermany; 4 n.a.Wissenschaftliches Institut für Gesundheitsökonomie und Gesundheitssystemforschung (WIG2 GmbH)LeipzigGermany; 5 Poliklinik für Zahnärztliche Prothetik und Werkstoffkunde 70622 Universität Leipzig Medizinische FakultätLeipzigSNGermany; 6 Institut für Medizinische Biometrie, Epidemiologie und Informatik | Abteilung Epidemiologie und Versorgungsforschung 39068 Universitätsmedizin der Johannes Gutenberg-Universität MainzMainzRPGermany; 7 BerlinTMF - Technologie- und Methodenplattform für die vernetzte medizinische Forschung e.V.BerlinGermany; 8 Poliklinik, Funktionsbereich & Hiller Forschungszentrum für Rheumatologie 39064 Universitätsklinikum DüsseldorfDüsseldorfNordrhein-WestfalenGermany; 9 Rheumatology 9170 Heinrich-Heine-Universitat DusseldorfDüsseldorfNRWGermany; 10 Zentrum für Digitale Medizin 9170 Heinrich-Heine-Universitat DusseldorfDüsseldorfNRWGermany; 11 CCC-MV, Lebensqualität in der Onkologie 39071 Universitätsmedizin RostockRostockMVGermany; 12 Medizinische Informatik 9171 Friedrich-Alexander-Universitat Erlangen-NurnbergErlangenBYGermany; 13 Zentrum für Evidenzbasierte Gesundheitsversorgung (ZEGV)Universitätsklinikum und Medizinische Fakultät Carl Gustav Carus an der TU DresdenDresdenGermany



Im oben genannten Artikel wurde folgende Erklärung zu potenziellen Interessenskonflikten ergänzt: Ursula Kramer berät als CEO der sanawork GmbH Firmen bezgl. Evaluation und Marktzugang von DiGA, DiPA. Die Korrektur wurde in der Onlineversion des Artikels ausgeführt am: 23.09.2026

